# Bispecific Antibodies in B-Cell Lymphomas: Mechanisms, Efficacy, Toxicity, and Management

**DOI:** 10.3390/medicina62020342

**Published:** 2026-02-08

**Authors:** Ádám Jóna, Dávid Tóthfalusi, Árpád Illés, Zsófia Miltényi

**Affiliations:** 1 Department of Hematology, Faculty of Medicine, University of Debrecen, Member of ERN-EuroBloodNet (European Reference Network on Rare Haematological Diseases), H-4032 Debrecen, Hungary; tothfalusi.david@med.unideb.hu (D.T.); illes.arpad@med.unideb.hu (Á.I.); miltenyi.zsofia@med.unideb.hu (Z.M.); 2 Doctoral School of Clinical Medicine, University of Debrecen, H-4032 Debrecen, Hungary

**Keywords:** bispecific antibodies, B-cell lymphomas, immune effector cell-associated neurotoxicity syndrome, cytokine release syndrome, antimicrobial prophylaxis

## Abstract

Bispecific antibodies represent a pivotal advancement in treating relapsed/refractory B-cell lymphomas, addressing unmet needs for patients with limited conventional options. This review examines CD20 × CD3 bispecific antibodies (BsAbs) like mosunetuzumab, epcoritamab, odronextamab, and glofitamab, which link malignant B-cells and T-cells, thus inducing targeted tumor lysis. These IgG-like molecules activate T-cells, triggering proliferation and cytotoxic molecule release, bypassing MHC presentation. These agents have received regulatory approval for the treatment of various B-cell lymphomas and exhibit substantial efficacy, with high overall and complete response rates in diffuse large B-cell lymphoma and follicular lymphoma. However, their use is associated with immune-related toxicities. Cytokine Release Syndrome, which is a systemic inflammatory response due to a cytokine surge, and Immune Effector Cell-Associated Neurotoxicity Syndrome, linked to endothelial activation and blood–brain barrier disruption, are critical concerns. This review details their mechanisms, grading, and management, including the use of tocilizumab and corticosteroids. Furthermore, BsAb therapy carries an elevated susceptibility to viral, bacterial, and opportunistic infections, often exacerbated by hypogammaglobulinemia. Expert recommendations for antimicrobial prophylaxis, including herpes and varicella zoster virus, pneumocystis, and immunoglobulin supplements are crucial for mitigating these risks. While BsAbs offer an “off-the-shelf” advantage, balancing their efficacy with comprehensive toxicity management is crucial for maximizing patient outcomes.

## 1. Introduction

Bispecific antibodies (BsAb) constitute a significant advancement in the management of B-cell lymphomas, offering novel immunotherapeutic strategies through the redirection of immune effector cells against malignant B-cells [1]. These therapies fulfill a critical, unmet clinical need among patients with relapsed/refractory (R/R) B-cell lymphomas, who often face poor prognosis with conventional treatments, marked by progressive resistance to anti-CD20 antibodies and chemotherapy and coupled with declining survival rates across successive lines of treatment [2,3,4]. For patients with R/R disease, particularly in diffuse large B-cell lymphoma (DLBCL), outcomes can be dismal, with median overall survival sometimes as low as 6–7 months and salvage chemotherapy and autologous stem cell transplantation achieving cure in only approximately half of these patients [5]. While CAR T-cell therapies have demonstrated efficacy, their use is often limited by accessibility, manufacturing delays, high costs, and treatment-related toxicities [6]. In this context, bispecific antibodies provide an “off-the-shelf” therapeutic possibility [7].

Several CD20 × CD3 bispecific antibodies have received regulatory approval, predominantly for R/R follicular lymphoma (FL) and DLBCL patients after two or more prior lines of systemic therapy [1,5,8]. This review will detail the structure and mechanism of action of CD20 × CD3 bispecific antibodies, discuss their approved indications, summarize the safety and efficacy profiles of key agents, and outline the clinical management of associated toxicities, including Immune Effector Cell-Associated Neurotoxicity Syndrome (ICANS) and Cytokine Release Syndrome (CRS), as well as infectious complications.

## 2. Bispecific Antibodies: Structure and Mechanism of Action

### 2.1. General Structure of Bispecific Antibodies

Bispecific antibodies are engineered molecular constructs serving as molecular bridges to recruit immune effector cells, like T-cells, into close proximity with target tumor cells [9,10,11,12,13]. This dual specificity is a hallmark of their therapeutic mechanism, allowing for novel immunotherapeutic strategies. Structurally, these therapeutics exhibit considerable diversity, broadly categorized into immunoglobulin G-like formats, which retain an Fc region, and Fc-free formats [10,14,15,16].

Many modern bispecific antibodies are designed with an IgG-like structure, often featuring a symmetrical architecture [17,18]. The inclusion of an Fc region in IgG-like bispecific antibodies is critical for several pharmacokinetic advantages, including enhanced stability, simplified purification processes, and an extended half-life in serum by binding to the neonatal Fc receptor [10,13,14,15,19,20,21,22]. However, the Fc domain may also engage effector functions, such as complement-dependent cytotoxicity and antibody-dependent cellular cytotoxicity, which can result in non-specific immune responses if not carefully managed. Therefore, the Fc domain is frequently engineered to be “silenced” or rendered inert to avoid these unwanted effects, while still preserving the benefits of stability and extended half-life [13,14,23].

Conversely, Fc-free bispecific antibodies, like BiTEs (bispecific T-cell engagers), are typically smaller in size. This reduced molecular weight often translates to better tissue penetration and potentially lower immunogenicity [14,15]. However, a trade-off for their smaller size and lack of an Fc region is a significantly shorter systemic half-life compared to their Fc-containing counterparts [14,15]. While a shorter half-life can be a disadvantage for maintaining sustained therapeutic levels, it can also be an intentional design choice, offering a safety advantage by allowing for prompt systemic elimination should adverse events occur, as seen with blinatumomab [12]. The engineering of these diverse formats is complex, often requiring meticulous molecular design to ensure optimal binding functionality and biophysical stability, and to overcome challenges like antibody chain mispairing [11,23]. For instance, glofitamab features a novel 2:1 tumor–T-cell binding configuration that confers bivalency for CD20 and monovalency for CD3, specifically designed to efficiently engage and redirect T-cells to eliminate malignant B-cells [24].

### 2.2. Mechanism of Action of CD20 × CD3 Bispecific Antibodies

CD20 × CD3 bispecific antibodies function by physically bridging CD20-expressing malignant B-cells and CD3-expressing T-cells, thus creating an artificial immune synapse [8,18,25,26,27,28,29]. This critical interaction brings the immune effector cells into close proximity with the target tumor cells, enabling a targeted immune response. Upon simultaneous engagement of CD3 on the T-cell and CD20 on the malignant B-cell, the bispecific antibody triggers the activation of the T-cell receptor signaling cascade [28]. This activation leads to a series of downstream events, including rapid T-cell proliferation and the release of various cytotoxic molecules and pro-inflammatory cytokines [26,28,30]. Specifically, activated T-cells secrete perforin and granzyme, which are crucial for inducing the apoptosis and subsequent lysis of the CD20-positive malignant B-cells [18,27,28,31,32,33]. Furthermore, T-cell activation also results in the secretion of cytokines, including interferon (IFN)-γ, tumor necrosis factor, interleukin (IL)-2, IL-6, and IL-10, which can enhance the anti-tumor function and contribute to the systemic inflammatory response [18,27]. This mechanism bypasses the need for conventional major histocompatibility complex (MHC) presentation, allowing T-cells to recognize and eliminate tumor cells independently of MHC-peptide complexes [29]. The potency of these antibodies, and the extent of T-cell activation and cytokine release, can be significantly influenced by factors such as the affinity of the CD3-binding arm [34]. This differentiates them from conventional CD20 monoclonal antibodies, which primarily rely on Fc-mediated effector functions [35].

## 3. Efficacy, Safety Profiles, and Approved Indications in B-Cell Lymphomas

### 3.1. Mosunetuzumab

Mosunetuzumab constitutes a pioneering humanized CD20 × CD3 bispecific antibody engineered on an IgG1 scaffold [36]. It is assembled using “knobs-into-holes” technology [26]. In patients with R/R FL after at least two prior therapies, a pivotal phase II trial demonstrated an overall response rate (ORR) of 80% and a complete response (CR) rate of 60% [4]. With a median follow-up of 28.3 months, the median duration of response was 35.9 months, and overall survival was 82.4% at 36 months [37]. A step-up dosing schedule in cycle 1 was implemented to effectively mitigate CRS, making events predominantly grade 1 or 2 [4,37,38]. Grade 3 or higher CRS occurred in approximately 2.2% of patients, and Immune Effector Cell-Associated Neurotoxicity Syndrome occurred in 4.4%. The incidence of potential ICANS remained low at 5%, predominantly comprising grade 1 events. Mosunetuzumab received conditional marketing authorization from the European Medicines Agency (EMA) and accelerated approval from the US Food and Drug Administration (FDA) for R/R FL following ≥2 prior systemic therapies [1,37].

Real-world data complements clinical trial results by revealing the sustained efficacy and safety profile of mosunetuzumab in B-cell lymphomas, with several studies using real-world evidence to assess its performance. One study used a Bayesian framework to integrate multiple real-world data sources to estimate long-term overall survival with mosunetuzumab among patients with follicular lymphoma receiving third- or later-line therapy (3L+). This analysis indicated that hazard patterns from real-world data sources corroborated a constant or linearly declining hazard pattern. For patients receiving 3L+ therapy for FL, the mean pointwise overall survival was estimated at 0.52 at 8 years. Bayesian extrapolations incorporating mosunetuzumab data subsequently produced median survival estimates of 11.6 to 17.0 years, with uncertainty reduced by 20% to 46% [39].

In another effort, a matching-adjusted indirect comparison was performed to compare outcomes between a mosunetuzumab clinical trial and a real-world cohort from the Lymphoma Epidemiology of Outcomes Consortium for Real World Evidence study in R/R FL patients. After weighting the LEO CReWE cohort to balance clinical characteristics with the trial, the observed overall response rates and complete response rates in the weighted LEO CReWE cohort were 73% (95% CI: 65–80%) and 53% (95% CI: 45–61%), respectively. These rates were numerically lower than those reported in the mosunetuzumab clinical trial, which showed ORR of 80% (95% CI: 70–88%) and CR of 60% (95% CI: 49–70%) [40].

### 3.2. Epcoritamab

Epcoritamab constitutes a full-length human IgG1 CD3 × CD20 bispecific antibody that has shown potent anti-tumor activity in preclinical studies [3]. In R/R B-cell lymphoma, including DLBCL and FL, it showed clinically meaningful activity. In R/R FL patients, high ORR (82.0%) and CR rates (62.5%) were observed at a median follow-up of 17.4 months. In DLBCL patients, ORR was 68% with 46% achieving CR at full doses of 12–60 mg, and at 48 mg, ORR was 88% with 38% CR [35]. Epcoritamab generally showed no grade ≥ 3 CRS or any-grade ICANS events in comparative studies, suggesting a potentially improved safety profile compared to other bispecifics [41]. The incidence of any grade CRS in pivotal trials ranged from 39% to 67%, with grade ≥ 3 CRS being less than 5% [42]. EMA was approved for FL and DLBCL and in R/R disease after two or more prior lines of therapy. It received breakthrough therapy designation from the FDA for R/R FL after two prior lines of therapy [41].

An inverse probability of treatment weighting analysis directly compared the outcomes of patients treated with epcoritamab within the EPCORE NHL-1 trial to those from real-world clinical practice cohorts receiving 3L+ treatments, utilizing data from a US-based electronic health records database [43]. This analysis involved 157 epcoritamab-treated patients with large B-cell lymphoma and 179 LBCL patients treated with chemoimmunotherapy.

Further solidifying the understanding of epcoritamab’s profile in broader clinical contexts are the long-term follow-up results from pivotal studies. For relapsed or refractory (R/R) large B-cell lymphoma, the pivotal EPCORE NHL-1 study reported long-term efficacy and safety with a median follow-up of 25.1 months for 157 patients, showing an overall response rate of 63.1% and a complete response rate of 40.1% [44]. Likewise, the 3-year follow-up from the EPCORE NHL-3 trial in Japanese patients with relapsed/refractory diffuse large B-cell lymphoma indicated durable responses, high rates of minimal residual disease negativity, and a safety profile consistent with prior observations [45].

Regarding safety, epcoritamab generally demonstrated no grade ≥ 3 CRS or any-grade ICANS events in comparative studies, suggesting a potentially improved safety profile compared to other bispecifics [41]. Pivotal trials reported the incidence of any grade CRS ranging from 39% to 67%, with grade ≥ 3 CRS being less than 5% [42]. There is an associated risk of infections, including bacterial, viral, and opportunistic infections, which is an important consideration in the real-world management of patients receiving epcoritamab [42,46]. These analyses collectively contribute to a more complete picture of epcoritamab’s performance outside of initial trial settings, supporting its use and management in various B-cell lymphomas.

### 3.3. Odronextamab

Odronextamab is a hinge-stabilized, fully human IgG4-based CD20 × CD3 bispecific antibody [47] and has demonstrated significant efficacy in both FL and DLBCL. In patients with R/R FL, odronextamab achieved high rates of deep and durable responses, with 73% of patients attaining complete response [2]. For patients with R/R DLBCL who had progressed after CAR T-cell therapy, the ELM-1 study demonstrated an overall response rate of 48% and a CR rate of 32% [48]. Further, the ELM-2 study, a phase 2 multicohort trial, reported an ORR of 52.0% and a CR rate of 31.5% in DLBCL patients, with median durations of response and CR being 10.2 and 17.9 months, respectively [49]. Regarding safety, CRS is reported as 57% in one study [50]. However, these events are predominantly low-grade and manageable, as noted in general for bispecific antibodies [42]. A comparative study indicated a grade ≥ 3 CRS incidence of 3.9% [41]. ICANS is less frequent; grade 3 or higher neurotoxicity occurred in two patients in one study [50], and a comparative study reported an ICANS rate of 0.8% [41]. The ELM-1 study specifically noted no cases of ICANS [48], while the ELM-2 trial reported one patient experiencing a grade 2 ICANS event [2]. Infections are also a notable concern, with infections occurring in 20% of patients in the ELM-1 study, including grade ≥ 3 infections in 20.0% of patients [48]. Discontinuation due to adverse events occurred in 16% of patients in the FL study [2]. EMA was approved for DLBCL and FL in R/R disease after two or more prior lines of therapy [42].

### 3.4. Glofitamab

Glofitamab is distinct with a 2:1 tumor–T-cell binding configuration [24]. In patients with R/R DLBCL who had received at more than two prior lines of therapy, fixed duration glofitamab monotherapy achieved a 39% CR rate at a median follow-up of 12.6 months, with 78% of CRs ongoing at 12 months [24]. This included patients who had previously received CAR T-cell therapy, where 35% achieved CR [24]. The most common adverse event was CRS (63%), with grade 3 or higher CRS in 4% [51]. Neurologic adverse events of grade 3 or higher occurred in 3% of patients [51]. Any grade CRS in pivotal trials was between 39% and 67%. Glofitamab was approved by the EMA for DLBCL in R/R disease after two or more prior lines of therapy [42] and was also approved globally for R/R DLBCL [8,52].

An Israeli retrospective real-world study of 35 patients with R/R DLBCL reported an overall response rate of 34% and a complete response rate of 14%. In this real-world cohort, efficacy was inferior to that observed in clinical trials, primarily attributable to the heavily pretreated nature of the patient population; treatment was prematurely discontinued in 86% of cases, mainly due to disease progression and infections among responding patients [53]. Another compassionate use study in Turkey involving 43 patients showed an ORR of 37% and a CR of 21% [54,55]. In this cohort, grade ≥ 3 adverse events occurred in 23% of patients, with CRS reported as grade 1–2 in 28.6% and grade 3–5 in 8.6% of patients; hematological toxicity was most frequent, one patient died of CRS, and two died due to glofitamab-related febrile neutropenia [55,56]. No ICANS events were detailed in these reports. These real-world observations highlight the “efficacy-effectiveness gap” where real-world outcomes may not fully mirror those from controlled clinical trials, with a manageable but notable safety profile including infections and CRS.

In Table 1, we compare the above-mentioned CD3 × CD20 bispecific antibodies.

## 4. Management of Immune-Related Toxicities

### 4.1. Cytokine Release Syndrome

#### 4.1.1. Mechanism of CRS

CRS is a significant acute toxicity associated with T-cell engaging therapies, like bispecific antibodies. It results from on-target effects where the binding of the BsAb activates T-cells and other immune and non-immune cells, leading to the extensive release of pro-inflammatory cytokines such as IL-6, IL-10, and IFN-gamma [57,58]. This initial activation and local expansion of T-cells trigger a hypersecretion of IFN-gamma and TNF-alpha [9,57]. These early cytokines, in turn, activate other innate immune cells, such as monocytes/macrophages and non-immune cells, including endothelial cells and dendritic cells [9,57].

**Figure 1:**
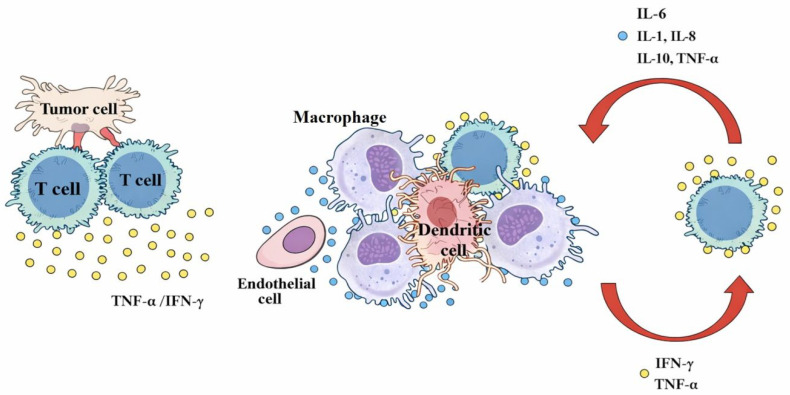
Adapted and modified from Shimabukuro-Vornhagen A., et al. [57], who report the mechanism of Cytokine Release Syndrome (CRS) when using bispecific antibodies in B-cell lymphomas.

The involvement of these bystander cells amplifies the inflammatory response. Macrophages, for instance, are a major source of IL-6 [9,57], a key cytokine driver in CRS [9]. Endothelial cells also contribute significantly to IL-6 production, particularly in severe CRS [57]. This massive and sustained release of cytokines establishes a positive feedback loop, where IL-6 further activates immune cells, leading to the uncontrolled escalation of the inflammatory process [57]. This surge of cytokines can overwhelm homeostatic mechanisms, resulting in a systemic inflammatory response, or “cytokine storm” [9,57]. A hallmark of severe CRS is the activation of endothelial cells, evidenced by elevated angiopoetin-2 and von Willebrand factor, which contributes to capillary leakage, hypotension, and coagulopathy, and may mechanistically link CRS to neurotoxicity (Figure 1) [57]. This widespread cytokine cascade leads to a constellation of symptoms, including chills, fever, fatigue, and anorexia. Severe cases can progress to organ dysfunction, such as cardiac and renal failure, or acute liver injury [9].

#### 4.1.2. Grading of CRS

The grading of CRS is performed according to the American Society for Transplantation and Cellular Therapy (ASTCT) consensus criteria, originally developed for CAR T-cell therapy and applicable to BsAb treatment [59]. The ASTCT grading system categorizes CRS based on clinical signs and symptoms, particularly fever, hypotension, and hypoxia, as listed in Table 2.

#### 4.1.3. Treatment of CRS

The management of CRS is led by its severity, as defined by the ASTCT consensus criteria [59] listed in Table 3. Treatment algorithms typically involve supportive care, anti-cytokine agents like tocilizumab, and corticosteroids, with interventions escalating based on the CRS grade [13,60]. Early intervention is crucial to mitigate severe outcomes [59].

In pivotal trials of bispecific antibodies for B-cell lymphomas, any-grade CRS occurred in 39–67% of patients (mostly grades 1–2, with grade ≥ 3 in <5%). CRS is most frequent during step-up dosing in cycle 1, often after the first dose of glofitamab or epcoritamab, and is typically low-grade with a predictable onset. Management typically begins with symptomatic measures for grade 1 CRS [42]. For more severe cases, treatment algorithms are based on expert opinion and may involve pausing BsAb infusion, corticosteroids, and IL-6 receptor inhibitors like tocilizumab [13,60]. BsAb treatment must be interrupted until resolution of CRS [42].

### 4.2. Immune Effector Cell-Associated Neurotoxicity Syndrome

#### 4.2.1. Mechanism of ICANS

Immune Effector Cell-Associated Neurotoxicity Syndrome (ICANS) is less common than CRS with bispecific antibodies but can occur, presenting as a serious and potentially fatal complication [42,65]. The precise mechanisms that underlie ICANS are complex and not yet fully elucidated, but they are intricately linked to cytokine-induced toxicity, profound endothelial cell activation, and the subsequent disorder of the blood–brain barrier (BBB) [66,67].

Following the systemic inflammatory response characteristic of CRS, a cascade of proinflammatory cytokines, including IL-1beta, TNF-alpha, IL-6, IL-15, IFN-gamma, and granulocyte–macrophage colony-stimulating factor, are released [65,68,69,70]. These circulating cytokines can directly impact the central nervous system (CNS), even from peripheral inflammation [71]. They induce injury and activate components of the BBB, such as astrocytes and pericytes, leading to further cytokine secretion and a breakdown of the barrier [67].

**Figure 2:**
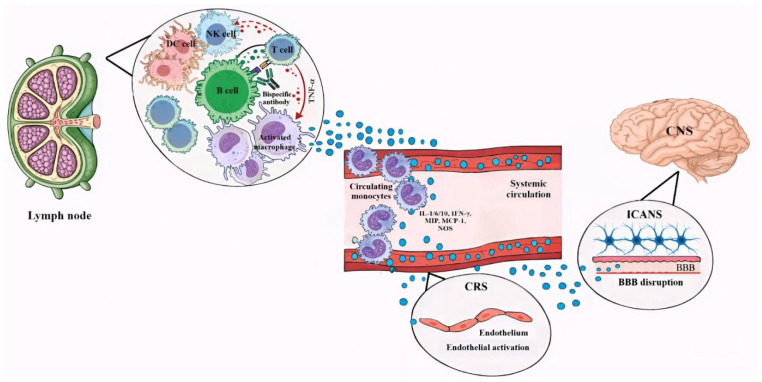
Mechanism of Immune Effector Cell-Associated Neurotoxicity Syndrome (ICANS), adapted and modified from Markouli M., et al. [9], when using bispecific antibodies in B-cell lymphomas.

Endothelial cell activation is a critical step in ICANS pathogenesis. This involves the release of angiopoietin-2 upon endothelial cell activation, which displaces angiopoietin-1 and results in increased endothelial activation and microvascular permeability [67,72]. This widespread systemic endothelial activation, often a consequence of the cytokine storm, promotes the upregulation of adhesion molecules (e.g., E- and P-selectin, ICAMs, VCAMs), leading to leukocyte activation and their migration into central nervous system tissue [73,74]. The resultant endothelial dysfunction and damage to tight junctions compromise the integrity of the BBB [67,70] (Figure 2).

The disruption of the BBB allows for the infiltration of large numbers of immune cells, inflammatory cytokines, and proteins into the CNS [67,70,72]. This influx exacerbates neuroinflammation, leading to various neurological symptoms. In severe instances, this process can culminate in cerebral edema, which may be profound and fulminant, and even intracerebral hemorrhage [66,67,68,75]. Neuroimaging often reveals cerebral edema in patients with severe neurotoxicity [64,76].

Clinically, adverse neurological events frequently manifest after the initial onset of CRS, suggesting that CRS can act as an initiating event or cofactor for ICANS [66,67,77]. It is common for ICANS to arise during the improvement or even resolution phase of CRS, though concurrent presentation can also occur [66,77]. ICANS clinical manifestations range from mild tremors and confusion to agitation, seizures, and severe encephalopathy [75].

#### 4.2.2. Grading of ICANS

ICANS is also graded using the ASTCT consensus criteria [59], as shown in Table 4. The frequency of any-grade ICANS in pivotal trials for BsAbs ranged between 0% and 8%, with most events being grade 1 and self-limiting [42].

#### 4.2.3. Treatment of ICANS

Treatment for ICANS involves a multi-pronged approach, varying based on the severity of the syndrome as graded by the ASTCT consensus criteria [59], as shown in Table 5. General strategies include corticosteroids, supportive care, and, in some cases, other immunomodulatory agents. Bispecific antibody treatment must be interrupted until ICANS is resolved [42].

## 5. Infectious Complications and Prophylaxis

### 5.1. Types of Infections Associated with Bispecific Antibody Use

Bispecific antibody therapy for B-cell lymphomas is associated with an increased risk of infections, including bacterial, viral, and opportunistic infections [42,46]. Risk factors for infection encompass hypogammaglobulinemia, cytopenias, and T-cell exhaustion [46]. Commonly reported severe infections include COVID-19, pneumonia, and sepsis [42]. Reactivation of viruses such as herpes simplex virus/varicella zoster, cytomegalovirus, and Epstein–Barr virus, as well as infections like *Pneumocystis jirovecii* pneumonia and fungal pneumonia, have been observed [42]. A high incidence of hypogammaglobulinemia (IgG < 400 mg/dL) is frequently observed, contributing significantly to infection risk [83].

### 5.2. Antimicrobial Prophylaxis Recommendations

Antimicrobial prophylaxis is crucial in managing infections associated with bispecific antibody use. Prophylaxis for *Pneumocystis jirovecii* pneumonia (PJP) and varicella zoster virus is recommended throughout BsAb treatment, continuing until CD4 counts recover to >0.2 × 10^9^/L [42]. For PJP, first-line agents include trimethoprim-sulfamethoxazole or inhaled pentamidine, with dapsone and atovaquone as alternatives [9]. VZV prophylaxis typically involves acyclovir, valacyclovir, or famciclovir for seropositive patients [9,82]. Antifungal prophylaxis is not routinely required [42] but should be considered in cases with additional risk factors, such as prolonged and severe neutropenia or following the administration of tocilizumab, anakinra, siltuximab, and/or high-dose steroids, continuing for at least one month after the last dose [9,84]. The routine administration of antibacterial prophylaxis in non-neutropenic patients remains controversial and is generally not recommended [9]. Nevertheless, in select high-risk scenarios or when the absolute neutrophil count declines below 500 mm^3^ for an extended duration, pharmacological prophylaxis is warranted pending neutrophil recovery, typically employing amoxicillin–clavulanic acid, levofloxacin, or cefdinir [9,82]. Intravenous immunoglobulin is recommended as the primary prophylaxis when IgG levels are <400 mg/dL, especially given the high rates of hypogammaglobulinemia [83]. It is also considered for patients experiencing recurrent or severe infections despite other antimicrobial prophylaxis, particularly with IgG levels < 4 g/L [42]. Typical IVIG dosing for prophylaxis in these settings is around 400 mg/kg every 2–4 weeks [82]. Prior to or at the initiation of BsAb therapy, patients should receive all routine non-live vaccinations, including those for COVID-19, influenza, and varicella zoster, while live vaccines must be avoided [9,42]. After immune reconstitution and B-cell aplasia recovery, re-vaccinations, including pneumococcal conjugated vaccines, are generally recommended [9]. Finally, BsAb treatment must be withheld in the presence of any active infection [42].

## 6. Conclusions

Bispecific antibodies have significantly improved the therapeutic options for B-cell lymphomas, offering effective options for heavily pretreated patients. These therapies demonstrate high efficacy, with overall response rates often approaching 80% and with complete response rates around 60%, varying with the specific agent and lymphoma subtype [5,35,85,86]. While effective, CAR T-cell therapies generally exhibit superior long-term efficacy [87,88]. Understanding their mechanisms, indications, and associated toxicities, along with robust management strategies for CRS, ICANS, and infectious complications, is paramount for optimizing patient outcomes.

Notably, while CRS and ICANS are potential adverse events, severe CRS (grade ≥ 3) is relatively rare with bispecific antibodies, occurring in 0–4% of patients [42]. In contrast, infectious complications pose a more significant challenge, with reported rates of infection between 40% and 80%, often being the most common cause of death [31,89]. This heightened infection risk is partly due to hypogammaglobulinemia, a frequent side effect of bispecific antibody therapy [90].

Bispecific antibodies are currently approved as monotherapy for R/R settings of B-cell lymphomas [85,91]. While they offer an “off-the-shelf” advantage over CAR T-cells—being standardized recombinant proteins that are immediately available for administration without the patient-specific manufacturing process required for CAR T-cells, which involves leukapheresis, genetic engineering, ex vivo expansion (typically taking 20–40 days), and risks of manufacturing failure (up to 10–25%) leading to potential disease progression during the wait [2,42,85]—their administration typically requires multiple, continuous doses. Although initial step-up doses often necessitate hospitalization for the close monitoring of adverse events, efforts are underway to facilitate outpatient administration for subsequent doses, particularly through subcutaneous delivery, to enhance accessibility and reduce healthcare burden [92,93]. These therapies represent a substantial financial investment, with costs for bispecific antibodies being significant, though often less than the upfront costs of CAR T-cell therapy [85,94]. However, the cumulative cost of bispecific antibodies over prolonged treatment durations, coupled with expenses for managing adverse events, can make their overall cost comparable to or even exceed a single CAR T-cell treatment [95,96].

Continued research into minimizing adverse events and improving prophylactic measures, particularly for infectious complications, will further enhance the safety and efficacy of these novel therapies.

## Figures and Tables

**Table 1:** Comparative table of CD20 × CD3 bispecific Antibodies.

Feature	Mosunetuzumab [4,37,38]	Epcoritamab [3,44]	Odronextamab [2,47,48]	Glofitamab [24]
**Structure/Mechanism**	Humanized CD20 × CD3 bispecific antibody (IgG1 scaffold), “knobs-into-holes” technology [26,36].	Full-length human IgG1 CD3 × CD20 bispecific antibody.	Hinge-stabilized, fully human IgG4-based CD20 × CD3 bispecific antibody.	2:1 tumor–T-cell binding configuration (bivalency for CD20 and monovalency for CD3).
**Approved indications**	R/R FL after ≥2 prior systemic therapies (conditional marketing authorization in EU, accelerated approval in US) [1,37].	R/R FL and DLBCL after $\ge$2 prior lines of therapy (EMA approval, FDA Breakthrough Therapy Designation for R/R FL) [41].	R/R DLBCL and FL in R/R disease after ≥2 prior lines of therapy [42].	R/R DLBCL after ≥2 prior lines of therapy [8,42,52].
**Efficacy**	R/R FL: ORR 80%, CR 60%. Median DoR 35.9 months, OS 82.4% at 36 months. Real-world ORR 73%, CR 53% [40]. Median survival estimates 11.6 to 17.0 years for 3L+ FL [39].	R/R FL: High ORR and CR rates [35]. R/R DLBCL: ORR 68% with 46% achieving CR (full doses); at 48 mg, ORR 88% with 38% CR [35]. Long-term EPCORE NHL-1: ORR 63.1%, CR 40.1%. EPCORE NHL-3 3-year follow-up: durable responses [45].	R/R FL: 73% CR. R/R DLBCL (post-CAR T): ORR 48%, CR 32%. R/R DLBCL: ORR 52.0%, CR 31.5%; median DoR 10.2 months, median CR 17.9 months [49].	R/R DLBCL: 39% CR (monotherapy, 12.6 months median follow-up); 78% of CRs ongoing at 12 months. 35% achieved CR in post-CAR T patients. Real-world ORR 34%, CR 14% [53]. Real-world ORR 37%, CR 21% [54,55].
**CRS**	Predominantly grade 1 or 2; grade ≥ 3 CRS in about 2.2%.	Any-grade CRS 39–67%; grade ≥ 3 CRS < 5% [42].	57% in one study (any grade); predominantly low-grade and manageable; grade ≥ 3 CRS 3.9% in comparative study [41,42].	Any-grade CRS between 39% and 67% in pivotal trials, mostly low-grade (grade 1 in 47% and grade 2 in 12%). Grade ≥ 3 CRS 4% in pivotal trials [51]; real-world 8.6% [55,56].
**ICANS**	4.4% (any grade); potential ICANS in 5%, predominantly grade 1; grade ≥ 3 not explicitly stated but implied low [4].	Generally showed no any-grade ICANS events and no grade ≥ 3 ICANS events in comparative studies [41].	Less frequent (any grade); ELM-1 noted no cases; ELM-2 reported one grade 2 event; Comparative study reported 0.8% (any grade); Grade 3 or higher neurotoxicity occurred in two patients in one study [41,48].	Not detailed in real-world reports (any grade); neurologic adverse events of grade 3 or higher occurred in 3% of patients [51].
**Infections**	Not explicitly stated in this section.	Associated risk, including bacterial, viral, and opportunistic infections [42].	20% in ELM-1 study, including 20.0% Grade ≥ 3 [48].	Observed in 38% of patients, with 15% experiencing grade ≥ 3 infections. Contributed to premature treatment discontinuation in 17% of patients (real-world) [53].

**Table 2:** Grading of Cytokine Release Syndrome (CRS) according to the American Society for Transplantation and Cellular Therapy (ASTCT) consensus criteria [59].

Measure	Grade 1	Grade 2	Grade 3	Grade 4
**Fever** *	≥38 °C	≥38 °C	≥38 °C	≥38 °C
**Hypotension**	No	No need for vasopressors	Need for a vasopressor with or without vasopressin	Need for numerous vasopressors (excluding vasopressin)
**Hypoxia**	No	Need for low-flow nasal cannula or blow-by	Need for high-flow nasal cannula, non-rebreather mask, facemask, or Venturi mask	Need for positive pressure support like CPAP, BiPAP. Intubation and mechanical ventilation

* Fever is defined as a body temperature ≥ 38 °C not attributable to any other etiology. In patients with CRS who subsequently receive antipyretic or anti-cytokine therapy (e.g., tocilizumab or corticosteroids), fever is no longer required for grading the severity of subsequent CRS episodes [59].

**Table 3:** Treatment of CRS as defined by the American Society for Transplantation and Cellular Therapy (ASTCT) consensus criteria.

CRS Grade	Treatment Recommendations
**Grade 1**	Symptomatic management with supportive care, including antipyretics and intravenous fluids. Monitoring can occur in a regular ward [60,61,62].
**Grade 2**	Supportive care and consideration of tocilizumab. Intensive monitoring and potential admission to an intermediate care ward or ICU should be considered [60,61,63]. Corticosteroids may be used, particularly if there is no adequate response to tocilizumab, or for older, co-morbid patients [62].
**Grade 3**	Aggressive supportive care, including intravenous fluids and pressor support for hypotension. Tocilizumab, with or without corticosteroids, is typically initiated. If refractory, or if deterioration occurs after initial treatment, higher doses of corticosteroids (e.g., dexamethasone or methylprednisolone) or other anti-cytokine agents (e.g., anakinra) may be considered. Patients require admission to the intensive care unit [9,60,64]. Bispecific antibody treatment must be interrupted until CRS resolution [42].
**Grade 4**	Similar to grade 3 but with more intensive supportive care, including mechanical ventilation if required. Persistent or severe cases may warrant further escalation of immunosuppression, potentially including additional anti-T-cell therapies (e.g., anti-TNF, ATG, Cy) [9,64]. BsAb treatment must be permanently discontinued for recurrent grade 3 CRS or grade 3 CRS lasting ≥48 h, or for any grade 4 CRS [9].

**Table 4:** Grading of Immune Effector Cell-Associated Neurotoxicity Syndrome (ICANS) according to the American Society for Transplantation and Cellular Therapy (ASTCT) consensus criteria.

Measure	Grade 1	Grade 2	Grade 3	Grade 4
Immune effector cell-associated encephalopathy (ICE) score	7–9	3–6	0–2	0 (patient is unconscious and not able to perform ICE) [59,78]
Level of consciousness	Awakens spontaneously	Awakens to voice	Awakens only to perceptible stimulus	Patient is unconscious or requires dynamic or repeated tactile stimuli to arouse; stupor or coma [59,78,79]
Seizure	No	No	Any clinical seizure (focal or generalized) that resolves rapidly or nonconvulsive seizures on EEG that resolve with intervention	Life-threatening prolonged seizure (>5 min) or repetitive clinical or electrical seizures without return to baseline in between [78]
Cerebral edema, increased intracranial pressure	No	No	Any edema on intracerebral imaging	Diffuse edema on cerebral imaging; decerebrate or decorticate posturing; or cranial nerve VI palsy; or papilledema; or Cushing’s triad [78]
Motor findings	No	No	No	Deep focal motor weakness such as hemiparesis or paraparesis [78]

Note: The ICANS grade is assigned according to the most severe event across the assessed measures [59]. The ICE score refers to the Immune-Effector Cell-Associated Encephalopathy score [80]. Depressed level of consciousness must not be attributable to any other cause [59,81].

**Table 5:** Treatment of Immune Effector Cell-Associated Neurotoxicity Syndrome (ICANS) as defined by the American Society for Transplantation and Cellular Therapy (ASTCT) consensus criteria.

ICANS Grade	Treatment	Other Recommendations
Grade 1	Vigilant supportive care; avoid aspiration; intravenous hydration [79]. Oral dexamethasone can be used [42]. Lorazepam (0.25–0.5 mg q 8 h, IV) or haloperidol (0.5 mg q6h, IV) for distressed patients [64].	Suspend oral intake of food, medications, and fluids; conduct a swallowing assessment. Transition all oral medications and/or nutritional support to intravenous administration if swallowing function is compromised [79]. If concurrent CRS, tocilizumab may be considered [64]. If agitated, haloperidol or lorazepam may improve symptoms [82].
Grade 2	Dexamethasone IV [42] (10–20 mg IV every 6 h [75,82]). Prophylactic antiepileptic, such as levetiracetam [42]. Methylprednisolone (1 mg/kg q12h) if refractory to anti-IL6 therapy [82]. Corticosteroids should be continued for a minimum of 48 h [79].	Other causes of neurotoxicity must be excluded (e.g., infection, opioid toxicity, medications, metabolic causes) [42]. If concurrent CRS, tocilizumab may be considered [64].
Grade 3	Dexamethasone intravenous [42] (10–20 mg intravenous every 6 h [75]). Anakinra [9,42]. Corticosteroids should be continued for 5–7 days with taper or until symptoms are resolved [79].	Managed in the intensive care unit setting [42]. Transfer to intensive care [75]. Exclude alternative etiologies [42]. May require high-dose methylprednisolone [42]. Consider alternative agents such as anti-TNF if unresponsive [82].
Grade 4	High-dose methylprednisolone [42] (e.g., 1 g intravenous methylprednisolone for at least 3 days [75]). Siltuximab [42]. Anakinra if unresponsive to high-dose methylprednisolone [82]. Corticosteroids should be continued for 5–7 days with taper or until symptoms are resolved [79].	Managed in the intensive care unit setting [42]. Transfer to the intensive care unit [75]. Mechanical ventilation may be necessary to ensure airway protection [9]. If the condition proves refractory, alternative agents such as anti-TNF inhibitors should be considered [82].

## Data Availability

No new data were created or analyzed in this study.
